# Elevated Angiopoietin-1 Serum Levels in Patients with Alzheimer's Disease

**DOI:** 10.1155/2012/324016

**Published:** 2012-10-10

**Authors:** Brigitte Schreitmüller, Thomas Leyhe, Elke Stransky, Niklas Köhler, Christoph Laske

**Affiliations:** ^1^Department of Psychiatry and Psychotherapy, University of Tübingen, 72076 Tuebingen, Germany; ^2^DZNE, German Center for Neurodegenerative Diseases, 72076 Tübingen, Germany; ^3^Section for Dementia Research, Department of Psychiatry and Psychotherapy, Hertie-Institute of Clinical Brain Research, University of Tübingen, 72076 Tübingen, Germany

## Abstract

*Background*. Alzheimer's disease (AD) is the most common cause of dementia in the elderly. AD is characterized by the accumulation of amyloid plaques and neurofibrillary tangles and by massive neuronal loss in the brain. There is epidemiologic and pathologic evidence that AD is associated with vascular risk factors and vascular diseases, contributing to cerebral hypoperfusion with consecutive stimulation of angiogenesis and upregulation of proangiogenic factors such as Angiopoietin-1 (Ang-1). *Methods*. In the present study, we measured Ang-1 serum levels in 42 patients with AD, 20 patients with mild cognitive impairment (MCI), and in 40 healthy elderly controls by ELISA. *Results*. We found significantly increased Ang-1 serum levels in patients with AD compared to control subjects (*P* = 0.003). There was no significant difference between MCI patients and healthy controls (*P* = 0.553) or between AD and MCI patients (*P* = 0.054). The degree of cognitive impairment as measured by the mini-mental status examination (MMSE) score was significantly correlated with the Ang-1 serum levels in all patients and healthy controls. *Conclusions*. We found significantly increased Ang-1 serum levels in AD patients. We could also show an association between Ang-1 serum levels and the cognitive status in all patients and healthy controls. Thus, serum Ang-1 could be a potential candidate for a biomarker panel for AD diagnosis.

## 1. Introduction

Alzheimer's disease (AD) is a progressive, irreversible neurodegenerative disease and the most common cause of dementia in the elderly [1]. Epidemiological studies have shown that risk factors for vascular diseases, including hypertension, diabetes, hypercholesterolaemia, hyperhomocysteinemia, and the apolipoprotein-4 genotype, are also important risk factors for AD, which indicate that their pathogenic mechanisms are connected [2]. AD patients also have more severe atherosclerosis in large cerebral arteries at the base of the brain (circle of Willis) than age-matched controls without AD [3]. Consecutively, cerebral blood supply is reduced in AD patients by atherosclerosis-induced vascular narrowing [4]. Reduced cerebral blood supply leads to cerebral hypoperfusion which is besides chronic inflammation one of the major clinical features in AD and could also play a critical role in its pathogenesis [5]. Cerebral hypoperfusion and consecutive hypoxia stimulate vascular activation and angiogenesis [6–8] and lead to the increase of adhesion molecules, cytokines and chemokines, such as Angiopoietin-1 (Ang-1) and vascular endothelial growth factor (VEGF). In addition, hypoxia may facilitate the pathogenesis of AD through a large number of cellular events leading to degenerative changes such as increasing amyloid beta (A*β*) generation, stimulating the hyperphosphorylation of tau, and impairing blood-brain barrier function [9–14].

Several growth factors or their lack have been implicated in the pathogenesis of AD. Angiopoietins are a family of growth factors specific for the vascular endothelium [15]. The specificity of the angiopoietins for the vascular endothelium results from the restricted distribution of the angiopoietin receptors, Tie1, and Tie2, to these cells. The four known angiopoietins all bind to Tie2, but it is still unclear as to whether they utilize the closely related receptor Tie1 [16]. Ang-1 is a 70 kDa glycoprotein which contains 498 amino acids, including an N-terminal secretory signal sequence. Two regions within the coding sequence display homology to myosin and the C-terminus of fibrinogen, respectively [15]. In the adult, Ang-1 is expressed at a low level in a wide range of tissues, acting as a maturation and stabilizing signal for mature vasculature [17]. Besides, Ang-1 seems to be important for the development of the vasculature [18]. To the best of our knowledge, there are no data in the literature describing a statistically significant difference between Ang-1 serum levels of AD patients, MCI patients, and healthy elderly controls.

The current study aimed to examine Ang-1 serum levels in AD patients, MCI patients, and healthy elderly controls and to examine the association with the degree of cognitive impairment as measured by the mini-mental state examination (MMSE).

## 2. Materials and Methods

### 2.1. Subjects

A total of 42 patients with AD, 20 patients with MCI and 40 healthy elderly controls, were included in the study. Baseline characteristics and demographic parameters are displayed in Table 1. AD and MCI patients were outpatients from our Memory Clinic of the Department of Psychiatry and Psychotherapy at the University Hospital of Tuebingen. Patients with AD fulfilled the criteria of ICD-10, DSM-IV, and the National Institute of Neurologic and Communicative Disorders and Stroke and the Alzheimer's Disease and Related Disorders Association (NINCDS-ADRDA) for probable AD [19]. Patients with MCI fulfilled the criteria of Petersen et al. [20]. The clinical severity of cognitive impairment was assessed by the MMSE [21]. The ethics committee of the University of Tuebingen approved the study and written informed consent that was obtained from each participant.

### 2.2. Blood Sampling

Peripheral venous blood was sampled into serum tubes between 08:00 and 09:00 hours (fasting state) in order to take in account a possible circadian rhythm. Tubes were immediately immersed in melting ice. To minimize the source of platelets, serum was centrifuged within 30 min after sampling and stored at −20°C until further analysis.

### 2.3. Measurement of Ang-1 Serum Concentration

Serum levels of Ang-1 were measured using an enzyme-linked immunosorbent assay (ELISA) kit according to the manufacturer's (R & D Systems, Wiesbaden, Germany) instructions.

### 2.4. Data Analysis

All statistical analyses were carried out using the statistical analysis software package SPSS 19 (SPSS, Munich, Germany). For comparisons of Ang-1 serum levels between subject groups (patients with AD, MCI, and healthy controls), we calculated a univariate ANOVA. The data are presented as mean ± S.D. Significance for the results was set at *P* < 0.05. We conducted a bivariate correlation analysis (Pearson correlation) between age, MMSE scores, and Ang-1 serum levels. The two-tailed *t*-test was used to assess differences between two groups in case of normal distribution. The Mann-Whitney *U*-test was used to assess differences between two groups in case of nonnormal distribution. The chi-square test was used to assess differences in gender between two groups.

## 3. Results

**Figure 1:**
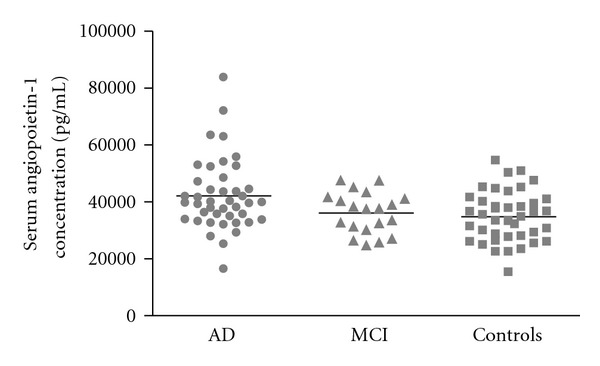
Angiopoietin-1 (Ang-1) serum levels (ng/mL) in Alzheimer's disease (AD) patients, mild cognitive impairment (MCI) patients, and healthy elderly controls. AD patients showed significantly higher Ang-1 serum levels compared with healthy controls (*P* = 0.003). There was no significant difference between MCI patients and healthy controls (*P* = 0.553) nor between AD and MCI patients (*P* = 0.054).

Accounting for the age difference between controls and AD patients, we included age as a covariate for the ANOVA. We found a difference in Ang-1 serum levels between the three different groups (*P* = 0.003). Pairwise comparisons with an independent *t*-test revealed a significant difference between AD patients and controls (AD versus healthy controls [mean ± SD] 42.1 ± 12.6 versus 34.8 ± 8.9 ng/mL; Figure 1). There was no significant difference between MCI patients and healthy controls (*t*(58)-0.596; *P* = 0.553) nor between AD and MCI patients (*t*(60) = 1.969; *P* = 0.054).

**Figure 2:**
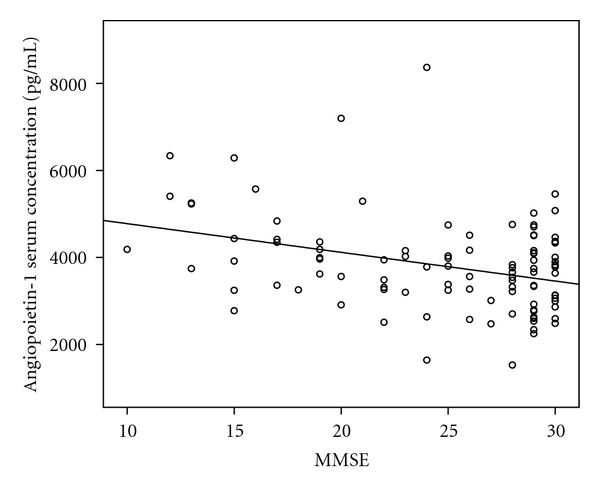
In all patients and healthy controls (*n* = 102) there is a significant correlation between the MMSE score (as a measure for cognitive status) and Ang-1 serum levels (*r* = 0.341; *P* < 0.001).

There is a significant positive correlation between the MMSE score (as a measure for cognitive status) and Ang-1 serum levels seen in all patients and healthy controls (*n* = 102) (*r* = 0.341; *P* < 0.001; Figure 2). There was no significant correlation between Ang-1 serum levels and MMSE score in AD patients (*r* = 0.23; *P* = 0.15).

## 4. Discussion

The major findings of the present study are as follows. (1) Ang-1 serum levels are significantly higher in AD patients compared to healthy controls. (2) Taking into account the confounding effect of altered Ang-1 serum levels and MMSE scores in AD patients, Ang-1 serum levels are significantly inversely correlated with MMSE as a measure for cognitive status in the whole study population. This indicates that a lower degree of cognitive functioning is associated with higher Ang1-serum levels.

Hypoperfusion of the brain caused by atherosclerotic changes of the vessels is assumed to play an important role in the pathogenesis of AD [5]. This hypoperfusion leads to hypoxia-induced angiogenesis via upregulation of hypoxia-inducible genes of Ang-1 and VEGF [8]. Together with VEGF, Ang-1 is capable of augmenting angiogenesis [22]. Coexpression of Angiopoietin-1 and VEGF prevents leakiness associated with VEGF alone [23]. Ang-1 acts as an antipermeability factor which is one of the most important biological roles of the angiopoietins. Vascular permeability is a fundamental component of the inflammatory response and therefore, Ang-1 also has an important anti-inflammatory role [24]. The elevated Ang-1 serum levels in patients with AD could be interpreted as a result of this hypoxia-induced angiogenesis. This is consistent with the findings that cerebral ischemia resulted in the induction of both Ang-1 and Ang-2 genes [25]. In the ischemic brain, expression of Ang-1 and VEGF is temporally and spatially correlated with neovascularization [26]. Another group was also able to show that transgenic overexpression of Ang-1 in the skin of mice produces larger, more numerous, and more highly branched vessels [27].

Angiogenesis is a complex process and consists of several discrete steps beginning with endothelial activation. Under normal conditions, endothelial activation is reversible and self-limiting. In AD, there is a continuous vascular activation induced by hypoperfusion, and factors and processes associated with angiogenesis can be found in the brain [28]. However, there is no evidence for increased vascularity in AD. On the contrary, there are several studies showing decreased microvascular density in the AD brain [29, 30]. One possible explanation could be that in response to a persistent stimulus such as cerebral hypoperfusion brain endothelial cells become activated and acquire an “activated angiogenic phenotype” [31]. No new vessels are formed which is the reason why there is no feedback signal to shut off vascular activation. The endothelial cells become irreversibly activated, and the products of the dysfunctional endothelium could injure or kill neurons [31].

Besides angiogenesis, inflammation plays another important role in the pathogenesis of AD [1]. Nevertheless, it is still not fully clear how and when inflammation arises in the course of AD, and the link between vascular inflammation, neuronal dysfunction, and death has not been clearly defined [32]. At the molecular level, inflammatory mediators are most highly expressed around A*β* deposits and neurofibrillary tangles in the brain from AD patients. There is evidence for inflammatory toxicity in the AD brain. For example, complement fixation and lysis of neurites can be demonstrated ultrastructurally [32, 33]. Several studies strongly suggested that conventional anti-inflammatory drugs may delay the onset or slow the progression of AD [32]. Under inflammatory conditions, there is also a pathological increase in vascular leakage, mediated, for example, by VEGF [34, 35]. In this context, Ang-1 seems to counteract VEGF-induced inflammation and vascular leakage in endothelial cells while having an additive effect on vessel formation [23]. Thus, the increase of Ang-1 serum levels in AD patients as demonstrated in the present study could be interpreted as an attempt of the human organism to encounter vascular inflammation and leakage seen in AD.

In conclusion, we found significantly increased Ang-1 serum levels in AD patients. We could also show an association between Ang-1 serum levels and the cognitive status in all patients and healthy controls. Thus, serum Ang-1 could be a potential candidate for a biomarker panel for AD diagnosis.

## Figures and Tables

**Table 1:** Patients' demographic and clinical details.

Characteristics	All (*n* = 102)	AD (*n* = 42)	MCI (*n* = 20)	Control (*n* = 40)	*P* value (AD versus controls)	*P* value (AD versus MCI)
Age (years)	69.9 ± 9.4	73.0 ± 8.0	67 ± 10.2	65.8 ± 8.8	0.001°	0.040
Sex (no. [%])					0.334*	0.597
Females	61 (59.8)	24 (57.1)	10 (50)	27 (67.5)		
Males	41 (40.2)	18 (42.9)	10 (50)	13 (32.5)		
Mini-mental state examination score (MMSE)	24.7 ± 5.6	19.2 ± 4.5	27.3 ± 1.9	29.2 ± 0.7	<0.001	<0.001

°Mann-Whitney *U*-test, *Chi-square test.
